# Diagnostic Yield and Performance of a Large Population-Based Cervical Cancer Screening Program in High-Risk Rural China

**DOI:** 10.7150/jca.41472

**Published:** 2020-04-06

**Authors:** Zhilian Wang, Tiannan Wang, Jing Yang, Wei Wang, Lili Zhang, Xiaoqiang Su, Zhe Wang, Haitao Zhang, Jinghui Song, Weiguo Lv, Jintao Wang, Chen Wang, Chengquan Zhao, Min Hao

**Affiliations:** 1 Department of Obstetrics and Gynecology, the Second Hospital of Shanxi Medical University, Taiyuan, China.; 2 Department of Pathology, Magee-Womens Hospital, University of Pittsburgh Medical Center. 300 Halket St, Pittsburgh, PA, USA.; 3 Department of Obstetrics and Gynecology, Affiliated Hospital of InnerMongolia Medical University, Huhhot, China.; 4 Department of Gynecologic Oncology, Women's Hospital, School of Medicine, Zhejiang University, Hangzhou, China.; 5 Department of Epidemiology, School of Public Health, Shanxi Medical University, Taiyuan, China.; 6 Department of Pathology, the Second Hospital of Shanxi Medical University, Taiyuan, China.

**Keywords:** Cervical cancer, Population based study, primary cytology screening, Cervical cancer diagnostic yield

## Abstract

Primary cervical cancer screening by liquid-based cytology alone or with adjunctive HPV testing are available worldwide. However, little if any information is available about cervical cancer diagnostic yield of population-based cervical cancer screening in China. In response to it, we conducted a large prospective study on 40,000 women cervical cancer screening within six-month period in rural Shanxi Province, where has been reported as the highest cervical cancer rates in China. A standard cross-sectional survey by self-completed questionnaire was collected and followed by the liquid-based cytology screening. Follow-up biopsy with the diagnosis of cervical intraepithelial neoplasia 2 or higher lesion (CIN2+) were analyzed. Of initial 40,000 women participating in this study, 6.76% (2702/40,000) women had ASC-US or higher cytology screening results with ASC/SIL ratio at 6.14 (2381:388). Among them, 1.96% (782/40,000) women were found CIN lesions (95% CI, 1.68-2.23%) on confirmatory biopsies, including 0.55% (218/40,000) CIN2+ and 19 SCCa (47.5/100,000, 95% CI, 29-74/100,000). Women in Yangqu County had lower ASC/SIL ratio, but higher CIN2+ detection rate in comparison with that of Jiexiu County (6.69 vs. 8.84 and 56.7% vs. 43.9%), which reflects the cervical cancer distribution in different populations and regions. Analysis in age-stratified cytology results indicated women aged 60-65 years had the highest incidence of cytologic abnormality among all the age group; likewise, women aged >50 years were at higher risk in developing cervical high grade dysplasia or cancer than women aged <50 years (0.82% vs. 0.49%). This large-scale cervical cancer screening study provided important references as the instructive for establishing the nation-wide cervical cancer screening strategy.

## Introduction

Papanicolaou (Pap) test as a revolutionary procedure in cervical cancer prevention has been implemented globally for more than 50 years 1, 2. According to the most recent Surveillance, Epidemiology, and End Results (SEER) data from 2012-2016, the incidence rate for cervical cancer had declined to 7.3/100,000 per year in the United States in contrast to 15.6/100,000 per year in China. Despite of local efforts and international assistance to scale up effective cervical cancer prevention service 3-5, about 80% cervical cancer burden from source-poor regions remains as a nationwide health issue 6-8. Latest cervical cancer statistical studies have shown that more than 85% of cervical cancer patient in Shanxi Province had never received proper screening and presented at late stage. As a result, the incidence and mortality of cervical cancer women living in Shanxi Province are 10-fold higher than that of other regions in China 9, 10. To date, little if any information is available concerning cervical cancer screening performance and diagnostic yield in this region 9-12. To address the issues, in this study, we aimed to evaluate the screening performance for CIN2+ lesion in a large Chinese female population of cervical cancer high risk region, Shanxi Province, to establish fundamental reference data. By doing that, we prospectively screened 40,000 married women from Jiexiu and Yangqu County of Shanxi Province by Pap tests, and characterized cytology screening data as well as follow-up biopsy diagnosis. We hope this large population based study could tremendously contribute to cervical cancer prevention in China.

## Materials and Methods

### Study Design and Population

Between June 2014 and November 2014, a total of 40,000 (31.8%, 40,000 / 126,000) women aged 18-to 65-year from 126,000 females of 109 villages in Jiexiu and Yangqu County in Shanxi Province were enrolled from total female population of 5,250,000 in Shanxi Province (2.4%, 126,000 / 5,250,000). The project was divided into two phases. Briefly, the first phase was to recruit women who voluntarily filled a standardized questionnaire, included sociodemographic and risk factors. Women were considered to be eligible if they: 1) Han ethnicity; 2) had lived in Jiexiu and Yangqu County for more than 1 year; 3) not being pregnant; 4) had not have the history of hysterectomy or conization; 5) had not have cervical physical therapy or treatment within the past one month. After the interview, 40,000 women were selected to undergo the cervical cancer screening by Pap test. The fieldwork, data collection, and readings were conducted by the expert panel of pathologists at Diagnostics Laboratory of Shanxi Medical University affiliated Second Hospital according to the Bethesda System (TBS). In the second phase, women with cytological results classified as ASC-US or higher on Pap test were referred to the colposcopic assessment. Negative / inadequate colposcopic findings after abnormal cytological results within 12-week warranted the endocervical curettage (ECC). Colposcopy directed biopsy on any suspicious area was taken and sent for pathologic diagnosis.

The procedure and the survey content were approved by the ethics committee of Second Hospital, Shanxi Medical University in China. The privacy were mostly insured during the interviews and procedures. All the protocols and the results are registered into the Chinese Clinical Trial Registrar (registration number: ChiCTR-ROC-15006479).

### Data Collection, Testing Methods and Analysis

#### CIN Questionnaire Survey and Cytology Specimen Collection

The questionnaire was adapted to reflect Shanxi Province population and relevant health issues of Shanxi residence. The questionnaire included primary independent variables such as household information, socioeconomics, and the direct variables such as menstrual, obstetrical history, sexuality reproductive conditions, and contraception, as well as other indirect variables such as medical history, and family history of cancer 10.

40,000 eligible women underwent the pelvic exam individually in six-month period and cervical swab sample were obtained simultaneously. All participants were instructed to abstain from sexual intercourse and not to perform vaginal lavage or take medications within 48-hours prior to the sampling. Repeat cytology was warranted for the cases with inadequate Pap cytological reports. All Pap slides were reviewed by two experienced cytopathologists. Cases with the diagnosis of ASC-US or higher were reviewed by the third cytopathologist. All the cytology interpretation were reported by adopting the 2001 TBS 13.

#### Colposcopy and Histopathologic Follow-up

Colposcopy visits were performed within twelve-week window upon the abnormal finding on Paps. During the procedure, entire cervix was carefully examined and all visually abnormal areas were biopsied. Random biopsies from squamocolumnar junction (transitional zone) were taken if absence of visible lesions. Endocervical curettages were proceeded in according to the aforementioned indications 14. Different gynecologic pathologists reviewed the biopsies and were blinded to the cytologic diagnosis 15. Review of results used in the present study were based on a consensus or the opinion of a third consultant pathologists.

#### Statistical Analysis

Descriptive statistics for the sociodemographic characteristics were entered into the database. Statistical Package for the Social Sciences (SPSS) (SPSS Inc, Chicago, IL, USA) were used for data analysis. Measurement data were expressed as means and standard deviation (Mean ± SD), and compared between groups by using analyses of variance. Enumeration date were expressed as rates, statistical analyses by the chi-square tests (χ^2^ test) for trend testing to compare the trend differences among the groups. The level of significance was set at p<0.05 (2-tiled). Given less than 20 SCCa cases were diagnosed on biopsy, 95% CI was determined through the look-up table method.

### Reagents and Instruments

A liquid-based cytology processor (Lituo Biotechnology Co.Ltd., China) and the LTS Liquid-based Thin-layer Cytology Smear Reagent Kit (Lituo Biotechnology Co. Ltd., China) were utilized for cytology specimen processing. SLC-2000 device (Shenzhen Goldway Company, Shenzhen, Guangdong, China) was utilized to perform video colposcopy procedure.

## Results

Of a total of 40,000 women recruited between June 2014 and November 2014, 255 (0.64%) had unsatisfactory Pap tests initially and underwent resampling, resulting in overall 6.76% (2702/40,000) ASC-US or higher Pap test reporting rate (Table 1.). Among them, ASC-US, ASC-H, LSIL and HSIL accounted for the reporting rate of 5.76%, 0.19%, 0.66% and 0.12%, respectively. Furthermore, abnormal Pap test reporting rate in Jiexiu County was statistically higher than the rate of Yangqu County (7.31% vs. 6.31%; p<0.001). Inversely, ASC-H reporting rate in Jiexiu County was much lower (0.04%, 7/20,000 vs. 0.35%, 70/20,000; p<0.01). Overall ASC/SIL diagnostic ratio was 7.71 (2381/309); it differed in Yangqu County (6.69, 1090/163) and Jiexiu County (8.84, 1291/146). Of note, 37,298 (93.25%) women were reported NILM; 2 (0.01%) SCCa and 10 (0.03%) AGC among 40,000 women were reported.

Age of participants largely affected cytology results. Table 2 presented the age-stratified cytology results by dividing women into five-age groups with ten-year gap of each group. Abnormal cytology reporting rate (ASC-US and higher) was significantly increased in the age-depended fashion (P<0.001). Of all of the age groups, women aged between 60 and 65 years had peak rate of abnormal Pap results in both Jiexiu (1438, 7.19%) and Yangqu County (1264, 6.23%), whereas women aged <30 years in both counties had least rate of abnormal Pap results with percentage of 2.34 and 2.48 respectively. Noticeably, women at age 50 -59 in both Jiexiu and Yangqu counties had sudden rise in abnormal cytology reporting rate comparing with women aged 40-49 years (12.94% vs. 5.42% and 11.26% vs. 4.38%).

**Figure 1:**
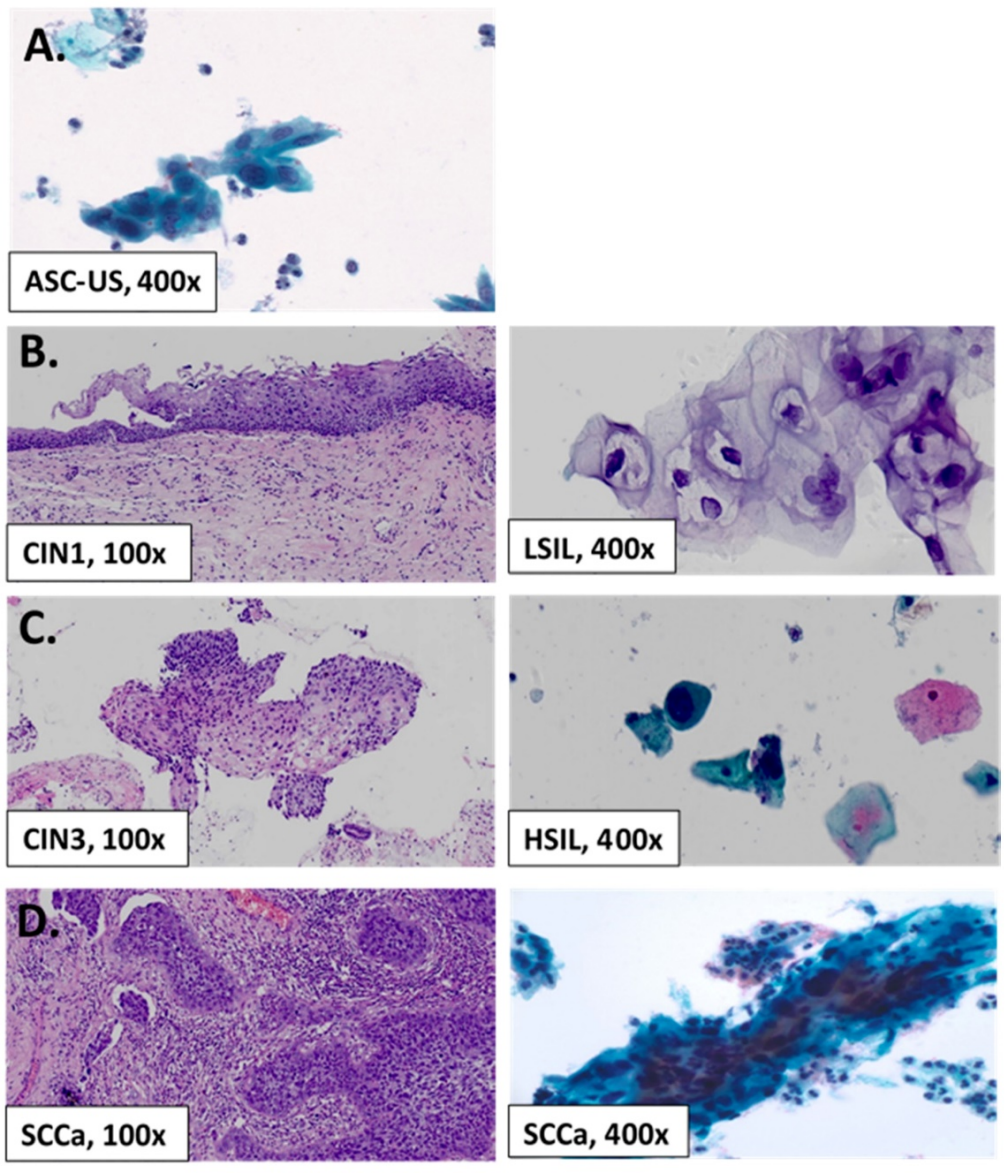
Representative Pap smear Slide Image and Follow-up Histology. (A) Example of ASC-US (400x) on liquid-based cytology. (B) Example of specimen cytologically diagnosed as low-grade intraepithelial lesion (LSIL, 400x) and corresponding CIN1 on follow-up biopsy. (C) Example of high-grade intraepithelial lesion (HSIL, 400x) on cytology and CIN3 on follow-up histology. (D) Example of cytologically diagnosed squamous cell carcinoma (SCCa, 400x) on cytology and SCCa on follow-up histology.

All but 2691 of 2702 (99.59%) women with abnormal Pap cytology had the histological follow-up, which were available for analysis (Table 3.), and the representative images of abnormal Pap cytology and follow-up biopsy histology were shown in Figure 1. 11 women with abnormal cytology refused to have colposcopic biopsy. CIN1+ lesions were diagnosed in 801, yielding an overall prevalence of 29.8%. In Jiexiu County, CIN1+ was detected in 421 women (2.11%, 421/20,000) including 317 CIN1 (2.05%), 93 CIN2/3 (0.47%), and 11 cases of SCCa (0.06%); whereas CIN1+ were detected in 380 (1.90%) women in Yangqu County including 247 CIN1 (1.24%), 125 CIN2/3 (1.25%), and 8 SCCa (0.04%).

More CIN2+ detection rate were higher in Yangqu County (10.61% vs. 7.23%; p<0.01) in contrast to Jiexiu County, whereas CIN1 detection rate were higher in Jiexiu county than that of Yangqu County (22.04% vs. 19.71%; p<0.01). In terms of PPV of ASC-US, it did not vary significantly between two counties despite of aforementioned higher ASC-US reporting rate in Jiexiu County. But HSIL PPV are much lower in Jiexiu County (39.29% vs. 57.89%). In Jiexiu County, over half of ASC-H (57.6%, 4/7) resulted in CIN2+ lesion, whereas 18.57% (13/70) ASC-H women of Yangqu County were detected CIN2+ lesion. Overall prevalence of CIN1, CIN2/3 and SCCa were 564 (1.41%), 218 (0.55%) and 19 (0.05%, 4.75/100,000), respectively.

In total, 2691 women with mean age of 49.2±9.1 years had histopathologic follow-up. 564 women with CIN1 diagnosis were at mean aged 49.4±9.0 years and 237 women with CIN2+ diagnosis were at mean age 48.7±9.2 years. Table 4 presented that CIN2+ detection rate increased with age (p<0.001). More than two third of CIN2+ women lesion were aged between 40 and 59 years (71.3%, 169/273). Accordingly, women aged <30 years had least CIN2+ detection rate (1.27%, 3/273). By statistics, there was significant variation in CIN2+ detection rates among each age groups (p< 0.001).

## Discussion

Cervical cancer is a preventable and treatable disease, which has a relatively long precancer stage, and takes 8 to 10-year window to develop to invasive cancer. Cervical cancer cytology screening has been approved as the most effective way to detect precancerous lesions and guide early cervical cancer treatment 1, 2. Starting from 2012, ASCCP guideline has recommended the strategy of HPV and cytology co-testing for cervical cancer screening in women aged 30-65 years 16, if combined with follow-up cervical biopsies for histologic confirmation as standard care of cervical cancer screening. However in reality, large-scale population-based screening approach by co-testing is costly, especially in developing countries and regions. On the other hand, women from high risk but resource-poor counties or regions necessarily demand for cervical cancer screening service. The pilot study on the population-based cervical cytology screening in such area would be instructive and referral.

### Cytology reporting rates differ in Yangqu County and Jiexiu County

To our best knowledge, this study is one of largest population-based prospective study on cervical cancer screening and cervical dysplasia diagnostic yield in China. 40,000 research subjects were recruited exclusively from the cervical cancer high-risk Jiexiu and Yangqu counties in Shanxi province within six months period. Overall abnormal cytological results was 6.76% (2702/40,000), including 5.76% (2304/40,0000) reported ASC-US, which was higher but consistent with the benchmark data of most recent College of American Pathologist survey (median rate: 5.6%; range: 1.1-13.3%) 17. The total reporting rate of combined ASC-H, LSIL, HSIL, AGC and SCCa was 1.01%, much lower than 7.1% reported by Pan et al 11. Likewise, AGC rate of both Yangqu and Jiexiu counties was only 0.03% (10/40,000), much lower than AGC rate in CAP survey in US population (median rate: 0.2%, range: 0-1%) and also lower than that of recent nationwide survey in China (median rate: 0.05%; range: 0-1%) 17,18. Similarly, Pan et al analyzed patients of community setting from other areas of Shanxi Province and reported 0.2-0.8% AGC rate, in which authors drew the conclusion of low incidence rate of cervical adenocarcinoma in Shanxi women population 11. But in fact, cervical gland cell abnormalities reporting rate in Chinese female population had been historically low at 0.01-0.06% 4, 19-21. Our data also showed ASC/SIL ratio of 6.14 (2381:388), which is outside the CAP range (0.8-5; median: 1.9), and higher than the data reported recently by the largest national survey (median: 2.1; range: 0.5-12) 18. The reasons for it are data elements in CAP report and other studies drew from limited opportunistic screening; by saying it, some patients may already present symptoms while seeking medical attention, resulting in high denominator in ASC/SIL ratio. Additionally, women in Yangqu County were found to have significantly lower ASC/SIL ratio compared with that of Jiexiu County (6.69 vs.8.84; p<0.01), predicting in difference in cervical cancer prevalence between Yangqu and Jiexiu populations. Individual program would have to consider the most suitable cutoff for triage based on regional needs. Likewise, LSIL rate in CAP survey was roughly 0.5 to 6, in contrast to 0.66% in our report, indicating LSIL reporting rate difference between Chinese and Western populations.

### Age dependent cytology reporting rate

Age distribution and sociodemographic factors may be the contributor to the difference in reports. This population based study found that the abnormal cytology reporting rates increased with age, which are consistent with cervical cancer prevalence in China. Overall CIN2 detection rate in women aged >50 was much higher than that of women aged <50 (0.82% vs. 0.49%; 100/12170 vs. 137/27830), similarly in CIN1 detection rate (2.53% vs. 0.92%, 308/12170 vs. 256/27830). In comparison of age distribution between Jiexiu County and Yangqu County, the peak age of abnormal reporting rate fall into 60-65 age group in both counties. Besides, 1565 abnormal cytology women in 50-65 age group accounted for 57.92% (1565/2702) of total abnormal participants. It has been debated that women over the age of 40 maybe start or experience the ovarian insufficiency, hormonal unbalanced, therefore tumor immunity microenvironment slowly decline. Nevertheless, it should be noted that Chinese women age-delay in reporting cytology abnormality reflects the current women health issue in rural China, such as low cervical cancer prevention awareness, delay in seeking timely medical attention and absence of standardized medical care.

### Diagnostic yield and performance of cervical cancer screening

As demonstrated by a population-based the study in Chinese population, ASC-US PPV was roughly 5% -17% 5. Accordingly, ASC-US PPV in present study were 6.28% in CIN2+, 19.97% in CIN1 and 0.57% in squamous cell carcinoma, similar to 6.5% (29/446) CIN2+ detection rate by Lopez-Alegria F et al.^22^. Other prospective studies had shown CIN2+ detection rate of 4.1% (107 / 2612) in Chinese population 11 and 8% in Mongolia population 12. Besides, it's worthwhile to mention that 19 biopsy-confirmed SCCa accounted for prevalence of 47.5/100,000 (95% CI, 29-74/100,000), surprisingly higher than 7.6/100,000 of 2011-2015 cervical cancer incidence rate of American Cancer Society statistics. Moreover, 95% CI at 29-74/100,000 for the cervical dysplasia detection rate is much higher than 9.6/100,000 in 2008 WHO, indicating the high incidence of cervical cancer in Shanxi Province 7. As such, we reinforced the necessity of high quality cervical cancer screening service in rural China and with no doubt, cytology screening is the cost-effective way to reduce the cervical cancer burden in this high risk region. Lastly, in the analysis of incidence rate by regions, of total 237 CIN2+cases among 40,000 women, 56.7% were from Yangqu County, in contrast to 56.5% of 564 CIN1 cases were from Jiexiu County. The fluctuations in detection rate in two counties may reflect the unbalanced cervical cancer distribution in different populations.

In the end, China still faces the huge challenges in the issue of cervical cancer prevention and treatment in rural female population 23. Our study looked at the six months cervical cancer screening data in a large population and helped to better understand the landscape of cervical cancer in women living in high risk Shanxi Province. In summary, our study showed 1.41% (564/40,000) CIN1 detection rate of 0.59% (237/40,000) CIN2+ detection rate and 47.5/100,000 SCCa in 31.75% of Shanxi female population. This prospective study also demonstrated women at age of 45-65 years old are the high risk group, and should mandatorily undergo cost-effective cervical cancer screening. Meanwhile, this large scale data is instructive for establishing the nationwide cervical cancer screening strategy. We reinforced that the results of population-based screening are reasonably different from the hospital laboratory data derived from opportunistic screening in some English literature.

## Figures and Tables

**Scheme 1:**
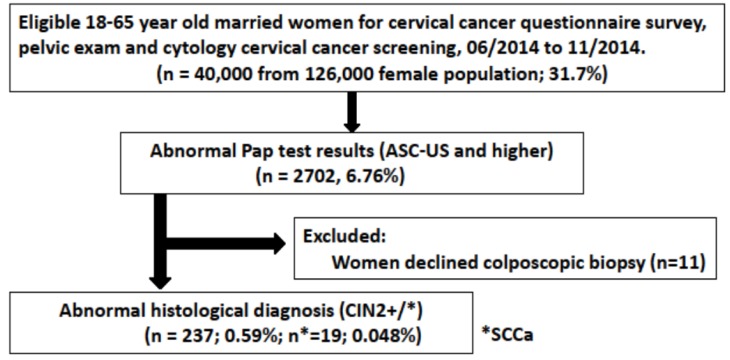
A flowchart to summarize the Recruitment of Study Population and Cyto-Histopathology Test

**Table 1:** Initial Cervical Cytological Screening Results By County (N=40,000)

County	ASC-US No. (%)	ASC-H No. (%)	LSIL No. (%)	HSIL No. (%)	AGC No. (%)	SCCa No. (%)	NILM No. (%)	Total
** Jiexiu**	1284 (6.42)	7(0.04)	118 (0.69)	28 (0.14)	2 (0.01)	2 (0.01)	18559 (92.80)	20,000
** Yangu**	1020 (5.10)	70 (0.35)	144 (0.72)	19 (0.10)	8 (0.04)	0 (0.00)	18739 (93.69)	20,000
**Total**	2304 (5.76)	77 (0.19)	262 (0.66)	47 (0.12)	10 (0.03)	2 (0.01)	37298 (93.25)	40,000

N, number; ASC-US, atypical squamous cells of undetermined significance; ASC-H, atypical squamous cells, cannot exclude high-grade squamous intraepithelial lesion; LSIL, low-grade squamous intraepithelial lesion; HSIL, high-grade squamous intraepithelial lesion; AGC, atypical glandular cells; SCC, squamous cervical carcinoma; NILM, negative for intraepithelial lesion or malignancy.

**Table 2:** Age-Stratified Cytology Results in Jiexiu County and Yangqu County (N=40,000)

	Jiexiu County		Yangqu County		Total	
**Age**	**Total Case** **No.**	**Abnormal Case** **No. (%)**	**Total Case** **No.**	**Abnormal Case No. (%)**	**Total Case** **No.**	**Abnormal Case** **No. (%)**
**< 30**	1497	35 (2.34)	1009	25 (2.48)	2506	60 (2.39)
**30-39**	4561	138 (3.03)	4555	148 (3.25)	9116	286 (3.14)
**40-49**	7799	423 (5.42)	8409	368 (4.38)	16208	791 (4.88)
**50-59**	5061	655 (12.94)	4964	559 (11.26)	10025	1214 (12.11)
**60-65**	1082	187 (17.28)	1063	164 (15.43)	2145	351 (16.36)
**Total**	20000	1438 (7.19)	20000	1264 (6.32)	40000	2702 (6.75)

N, number.

**Table 3:** Histological Follow-Up in Women with Abnormal Cytology by County (N=2,691)

	Jiexiu County			Yangqu County			Total		
**Pap Results**	**Total** **No.**	CIN2+/* No. (%)	**CIN1** **No. (%)**	**Total** **No.**	CIN2+/* No. (%)	**CIN1** **No. (%)**	**Total** **No.**	CIN2+/* No. (%)	**CIN1** **No. (%)**
**ASCUS**	1281	67/9* (5.23)	269(21.18)	1012	77/4* (6.40)	189(18.48)	2293/13*	144/13* (6.28)	458 (19.97)
**ASC-H**	7	4 (57.14)	3 (42.86)	70	13/3* (18.57)	19 (27.14)	77/3*	17/3* (22.08)	22 (28.57)
**LSIL**	118	20 (16.95)	37 (25.69)	144	32 (22.22)	37 (31.36 )	262	52 (19.85)	74 (28.24)
**HSIL**	28	11 (39.29)	8 (28.57)	19	11/1* (57.89)	2 (10.53)	47	22/1* (46.81)	10 (21.28)
**AGC**	2	0 (0.00)	0 (0.00)	8	0 (0.00)	0 (0.00)	10	0 (0.00)	0 (0.00)
**SCCa**	2	2/2* (100.00)	0 (0.00)	0	0 (0.00)	0 (0.00)	2/2*	2/2* (100)	0 (0.00)
**Total**	1438	104/11* (7.23)	317 (22.04)	1253	133/8* (10.61)	247(19.71)	2691/19*	237/19* (8.81)	564 (20.96)

N, number; ASC-US, atypical squamous cells of undetermined significance; ASC-H, atypical squamous cells, cannot exclude high-grade squamous intraepithelial lesion; LSIL, low-grade squamous intraepithelial lesion; HSIL, high-grade squamous intraepithelial lesion; AGC, atypical glandular cells; SCC, squamous cervical carcinoma; NILM, negative for intraepithelial lesion or malignancy; CIN, cervical intraepithelial neoplasia. *SCCa.

**Table 4:** Age-Stratified Histopathologic Follow-up (N=2,691)

Age	Negative No. (%)	CIN1 No. (%)	CIN2+ No. (%)		Total No.	
**< 30**	36 (19.04)	19 (3.37)	3 (1.27)		58	
**30-39**	184(9.74)	62(10.99)	39 (16.46)		285	
**40-49**	517(27.35)	175(31.03)	95 (40.08)		787	
**50-59**	915(48.41)	232(41.13)	74 (31.22)		1221	
**60-65**	238 (12.59)	76(13.48)	26 (10.97)		340	
**Total**	1890(100)	564(100)	237 (100)		2691	

*p*< 0.001. N, number; CIN, cervical intraepithelial neoplasia.

## Abbreviations

ASC-US+: atypical squamous cells of undetermined significance or worse
HSIL: high-grade intraepithelial lesion
LSIL: low-grade intraepithelial lesion
AGC: atypical glandular cell
SCCa: squamous cell carcinoma
CIN: cervical intraepithelial neoplasia
CIN2+: cervical intraepithelial neoplasia grade 2 or worse
CIN3+: cervical intraepithelial neoplasia grade 3 or worse
ECC: endocervical curettage
Pap test: Papanicolaou test
PPV: positive predictive value
TBS: The Bethesda System
